# Comparative analysis of the transcriptomes of two rice subspecies during domestication

**DOI:** 10.1038/s41598-021-83162-8

**Published:** 2021-02-11

**Authors:** Hongbo Pang, Qiang Chen, Yueying Li, Ze Wang, Longkun Wu, Qingwen Yang, Xiaoming Zheng

**Affiliations:** 1 College of Life Science, Shenyang Normal University, Shenyang, 110034 China; 2 Experimental Teaching Center, Shenyang Normal University, Shenyang, 110034 China; 3 Center for Crop Germplasm Resources, Institute of Crop Sciences, Chinese Academy of Agricultural Sciences, Beijing, 100081 China

**Keywords:** Genetics, Molecular biology, Plant sciences, Gene ontology

## Abstract

Two subspecies of rice, *Oryza sativa* ssp. *indica* and *O. sativa* ssp. *japonica*, with reproductive isolation and differences in morphology and phenotypic differences, were established during the process of rice domestication. To understand how domestication has changed the transcriptomes of the two rice subspecies and given rise to the phenotypic differences, we obtained approximately 700 Gb RNA-Seq data from 26 *indica* and 25 *japonica* accessions, and identified 97,005 transcribed fragments and 4579 novel transcriptionally active regions. The two rice subspecies had significantly different gene expression profiles, we identified 1,357 (3.3% in all genes) differentially expressed genes (DEGs) between *indica* and *japonica* rice. Combining existing gene function studies, it is found that some of these differential genes are related to the differentiation of the two subspecies, such as grain shape and cold tolerance, etc. Functional annotation of these DEGs indicates that they are involved in cell wall biosynthesis and reproductive processes. Furthermore, compared with the non-DEGs, the DEGs from both subspecies had more 5′flanking regions with low polymorphism to divergence ratios, indicating a stronger positive selection pressure on the regulation of the DEGs. This study improves our understanding of the rice genome by comparatively analyzing the transcriptomes of *indica* and *japonica* rice and identifies DEGs those may be responsible for the reproductive isolation and phenotypic differences between the two rice subspecies.

## Introduction

In addition to being a staple food that feeds over 50% of the world’s population, rice has also been used as a model plant for molecular, genetics and comparative genomic studies^1,2^. Rice varieties are traditionally classified into two major subspecies, *Oryza sativa* ssp. *indica* and *O. sativa* ssp. *japonica*, during the long process of rice domestication. These two rice subspecies are different in terms of yield, grain quality and stress resistance^2–5^. Indica rice is mainly cultivated in tropical and subtropical regions, whereas *japonica* rice is planted mainly in temperate regions or at higher altitudes in tropical and subtropical regions. During the process of domestication and breeding, these two subspecies have evolved characteristic morphological and agronomic traits those may contribute to intraspecific phenotypic adaptations^6,7^. The morphological features, including leaf color, seed size and apiculus hair length, cannot be used alone to definitively distinguish between the two subspecies due to the presence of morphological variants^8–10^. According to a previous report, multiple hybridization events between *japonica* and *indica*, both before and after the divergence of the *indica* cultivars^11^. However, the molecular mechanisms underlying the reproductive isolation and phenotypic differences remain largely unknown.

Thus far, there have been many studies on the molecular basis of the phenotypic differences between the two subspecies using different methods^12,13^. Among the methods used to search for candidate genes from hybrids of *indica* and *japonica* rice, the most important one is the quantitative trait locus (QTL)^14,15^. Using this method, several studies have managed to isolate and characterize candidate genes those are expressed in key tissues or at certain developmental stages from the two subspecies, indicating that gene expression variations contribute greatly to the phenotypic differences between the two subspecies^16–21^. Although there have been a number of transcriptomic studies and microarray analyses in several rice varieties, currently there are no transcriptomic studies carried out with multiple rice genotypes to establish a statistically significant genotype–phenotype correlation between two subspecies^22^. It has been suggested that nucleotide diversity in gene regulatory regions has a major impact on the expression of genes associated with the phenotypic differences between the two subspecies. In addition, it has been speculated that these gene regulatory regions may have evolved under positive selection pressure^23–26^. Although the gene-coding regions have been compared between *indica* and *japonica* rice, there is no method to effectively investigate the gene regulatory regions.

Studies on gene expression profiles and gene regulatory regions are limited by the high cost and inherent cloning bias of conventional high-throughput sequencing approaches and incomplete cDNA or expressed sequence tag (EST) libraries^19^. With the high sequencing efficiency and quality of paired-end-tag next generation sequencing, we are able to obtain large amounts of RNA-Seq data from a large number of samples and use these data to investigate gene expression profiles and study gene regulatory regions^27–32^. In this study, we analyzed the transcriptomes in the young panicle of 26 *indica* and 25 *japonica* accessions using RNA- Seq and identified 97,005 transcribed fragments and 4579 novel transcriptionally active regions. On this basis, we compared the transcriptomes of the two subspecies and identified 1357 differentially expressed genes (DEGs) between *indica* and *japonica* rice. Some of the identified DEGs were then confirmed by reverse transcription polymerase chain reactions (RT-PCR). This study has the following conclusions: (1) we report that the two rice subspecies have 1357 different gene expression profiles; (2) we propose the relationship between the DEGs and the phenotypic differences, including grain shape, cold tolerance and nitrate-absorption; and (3) we speculate that nucleotide diversity in gene regulatory regions may associate with the phenotypic differences and play an important role in crop domestication. Collectively, the results of this study improve our understanding of the rice genome.

## Results

### High-throughput transcriptome sequencing and read mapping in two rice subspecies

**Figure 1:**
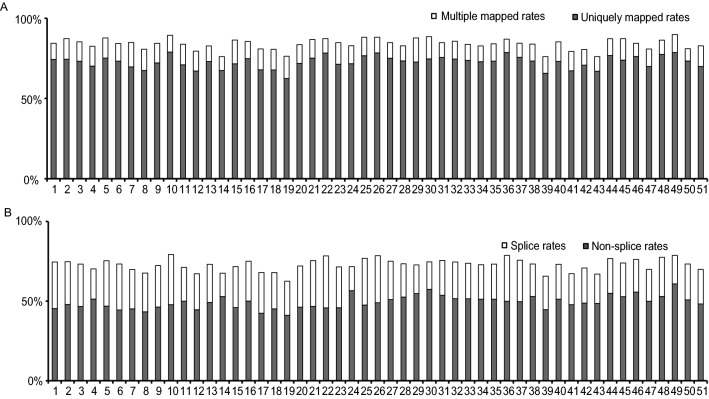
The mapping rates of the clean data in 26 *indica* and 25 *japonica* plants. (**A**) The mapping rates of the clean data. The white block represents the rate of multi-mapped reads, and the grey block indicates the rate of uniquely mapped reads. (**B**) The splice rate in 51 rice plants. The white block represents the splice rate, whereas the grey block indicates the non-splice rate. The numbers associated with the x-axis correspond to the sample name (see Table S1 for sample information). This figure was created using Microsoft Excel 2013 and Adobe Illustrator CS6.

To characterize the gene expression profiles at the reproductive stage of the two rice subspecies, we extracted total RNA from 40-mm-long young panicles of 26 *indica* and 25 *japonica* accessions (Table S1). For each plant, equal amounts of total RNA isolated from three panicles was mixed to establish an RNA pool, which was then further processed using a previously described method^33^ with minor modifications, and the samples were subjected to shotgun sequencing by an Illumina GAII instrument. We obtained 2.45 billion reads for the 26 *indica* accessions and 2.41 billion reads for the 25 *japonica* accessions at a 125 base-pair resolution (Table S2). After filtering out low-quality reads and reads containing adaptor sequences (7% of all reads), we obtained approximately 4.52 billion clean reads (345.48 and 331.99 Gb for *indica* and *japonica* accessions, respectively) (Table S2). These clean reads were then mapped to the reference *japonica* rice genome (Nipponbare; IRGSP v7.0), with at most two mismatches allowed for each read, while ignoring the reads that were mapped to more than two locations in the reference genome (multi-mapped reads). According to these criteria, we filtered out (~ 15%) of the clean reads and mapped 70.36–89.88% of the clean reads to the reference genome (Fig. 1A; Table S3). Among the mapped clean reads, 62.47–78.92% were mapped uniquely to one genomic location (Table S3). In addition, the rates of non-splice genes were 41.08–56.60% for *indica* accessions and 44.68–60.83% for *japonica* accessions (Fig. 1B; Table S3). We then calculated the mapping rates for each sample and the average mapping rates for the two subspecies. The results showed a significant difference in the average mapping rate between the two subspecies. The average mapping rate for *indica* accessions was 77.66%, whereas that for *japonica* was 83.88% (*t* = 19.275 and *p* < 0.001). Due to the fact that we used the *japonica* genome as the reference, we suspect that this difference may have been caused by mapping biases. Therefore, we decided to construct a pseudo-transcriptome of *indica* rice referring to the method in the tomato^34^ as the reference genome for the reads obtained from the *indica* rice samples. As a result, there was no significant difference in the average mapping rate between the two subspecies. The newly calculated average mapping rate for *indica* accessions was 84.01%, whereas that for *japonica* remained 83.88% (*t* = 0.12 and *p* = 0.91).

### RNA-Seq read assembly and identification of novel transcriptionally active regions

**Figure 2:**
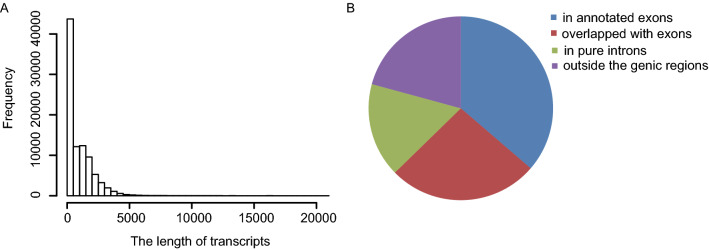
Summary of the assembled transcribed fragments. (**A**) Length distribution of the assembled transcribed fragments. (**B**) Classification of the assembled transcribed fragments. The transcribed fragments overlapping with annotated exons, exons, introns and non-gene regions are shown in blue, green, red and purple, respectively. This figure was created in Microsoft Excel 2013 and Adobe Illustrator CS6.

We subsequently assembled the RNA-Seq clean reads into 97,005 transcribed fragments with a mean length of 884 bp (ranging from 50 to 20,674 bp) (Fig. 2A). These fragments were aligned to the sequences of known rice genes (MSU 7.0). The sequence alignment results are shown in Fig. 2B. Among the assembled fragments, 26.31% overlapped with exons, 36.31% fell into annotated exons, 16.57% fell into introns, and the remaining 20.77% were non-gene sequences (Fig. 2B). We also found that 42% of rice genes had alternative transcripts. This percentage was much lower than that previously estimated based on microarray results^18^.

In total, we identified 4,579 novel transcriptionally active regions based on the sequences of rice genes and non-coding RNAs (ncRNAs) deposited in the Ensemble ncRNA and National Center for Biotechnology Information (NCBI) EST databases (E-value < 1e-6, Table S4). Among these regions, 61.7% were unknown protein-coding regions, 67.2% could be transcribed into single-exon transcripts, 42.71% shared more than 90% sequence identity with at least one known EST, and 12.14% were likely to encode non-coding RNAs. In addition, our RNA-Seq data expanded the untranslated regions (UTR) of 11.23% of rice genes.

### Identification of DEGs between the two rice subspecies

**Figure 3:**
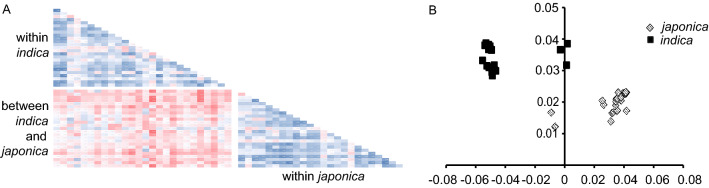
Significant differences in gene expression levels between *indica* and *japonica*. (**A**) Linear relationships in gene expression level between each sample. The R2 ranges from 0.75 (red) to 0.95 (blue). (**B**) The plot for principal variance component analysis of expression variations between *indica* and *japonica* plants. This figure was created using Microsoft Excel 2013 and Adobe Illustrator CS6.

According to the MSU rice genome annotation database, 74.71–88.49% of the mapped reads overlapped with annotated genes for each sample. The number of reads mapped to a certain annotated gene ranged from 2 to more than 300,000, with a median number of 23 and 32 for *indica* and *japonica* accessions, respectively. There were 40,580 genes mapped by two reads in at least two samples. In addition, we were able to establish linear relationships between the number of reads mapped to a certain gene and the gene expression level (FPKM) in all the samples (0.77 < R^2^ < 0.94; Fig. 3A). Given that we found significant differences in the number of reads mapped to a certain gene between the *indica* and *japonica* accessions (*t*-test; *P* = 0.012). Furthermore, principal variance component analysis also revealed significant differences in gene expression levels between *japonica* and *indica* individuals (Fig. 3B).

**Figure 4:**
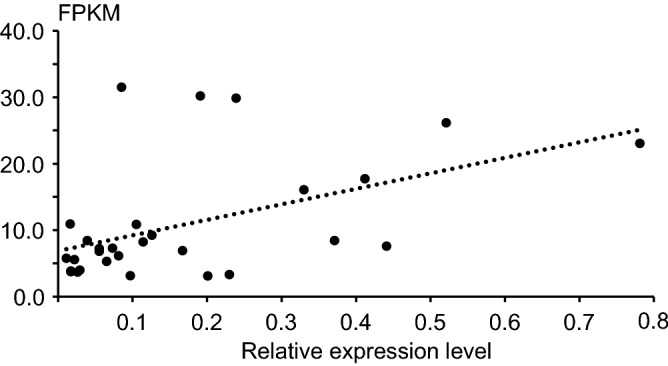
Correlation between qPCR results and FPKM values for 29 genes in the rice individuals. Each point represents the relative expression level of a gene. This figure was created in Microsoft Excel 2013 and Adobe Illustrator CS6.

**Figure 5:**
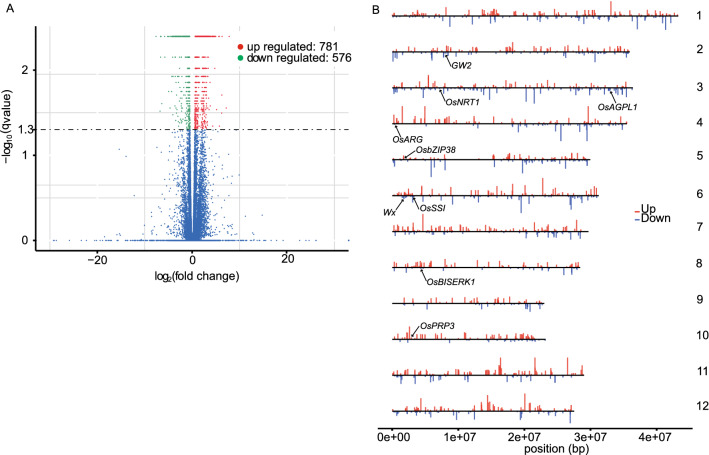
Summary of DEGs between *indica* and *japonica* rice. (**A**) Genes that were expressed at significantly different levels between *indica* and *japonica* rice. Red dots represent the up-regulated genes and green dots show the down-regulated genes. The blue dots represent non-DEGs. The volcano spots show 1357 unigenes, including 781 up-regulated unigenes and 576 down-regulated unigenes, which were identified as DEGs. (**B**) Distribution of the DEGs along rice chromosomes. The maps (A, B) were created in R (v.3.2.1; https://www.r-project.org/).

We then employed fragments per kilobase of transcript per million mapped reads (FPKM) values to estimate the expression level of various genes. To further confirm gene expression validation, 29 genes with different expression patterns revealed by RNA-seq were randomly selected for validation by quantitative real-time RT-PCR (qPCR). Three technical replicates were performed for each gene, and their specific primers were listed in Table S5. All of the 29 selected genes, only three genes were inconsistent with the results by the Illumina sequencing technology (Fig. 4). Taken together, most of the genes showed consistent expression patterns those were consistent with the RNA-Seq data, indicating that our experimental results were valid. Among the 41,080 expressed genes, 38,911 (94.72%) were expressed in both rice subspecies, 623 genes were expressed only in *indica* accessions and 1,546 genes were expressed only in *japonica* accessions. Thereafter, we analyzed our RNA-Seq data using a generalized linear model and identified 1,357 (3.3% of all the expressed genes) DEGs between *indica* and *japonica* rice (false discovery rate [FDR] < 0.05; Fig. 5A). 576 and 781 of these DEGs were expressed at lower and higher levels in *indica* rice than in *japonica* rice, respectively. (Fig. 5A). To identify biological functions of the differentially expressed genes (DEGs) (Table S6), we performed a literature search and found nine DEGs with important known functions including *GW2* (*LOC_Os02g14720*) and *OsBISERK1* (*LOC_Os08g07760*) that regulate grain shape in rice^13,35^. These two genes exhibit the same trend of expression changes, with higher expression levels in *indica* rice. *OsARG* (*LOC_Os04g01590*) and *OsNRT1* (*LOC_Os03g13274*), which are involved in nitrate (N) and ammonium absorption in *indica* rice^36,37^, may be related to the higher uptake of N in *indica* rice. One leucine zipper protein OsbZIP38, and Proline-rich protein OsPRP3, enhance cold stress responses in *japonica* rather than in *indica* rice^38,39^. The *Wx* (*LOC_Os06g04200*) gene encodes a granule-bound starch synthase (GBSS) and plays a key role in determining rice eating and cooking qualities (ECQs). In our results, the expression level of *Wx* in *indica* rice was up to 3-folds higher than in *japonica* rice, as well as *OsSSI* (*LOC_Os06g06560*) and *OsAGPL1* (*LOC_Os03g52460*) genes, which is consistent with the observation that the swelling of *indica* is stronger than *japonica* rice. We then mapped these DEGs to the rice genome and found they are distributed throughout the 12 chromosomes (*P* > 0.05; Fig. 5B). For better presentation of the results, these eight genes also be mapped on Fig. 5B. Furthermore, using REEF software (sliding window size was set to 500 kb to 1 Mb and the step size was set to 10 kb), we found that the DEGs tended to group in close proximity along the chromosomes (FDR < 0.05)^40^. However, we could not conclude that the DEGs were clustered.

### Functional annotation of the DEGs

**Figure 6:**
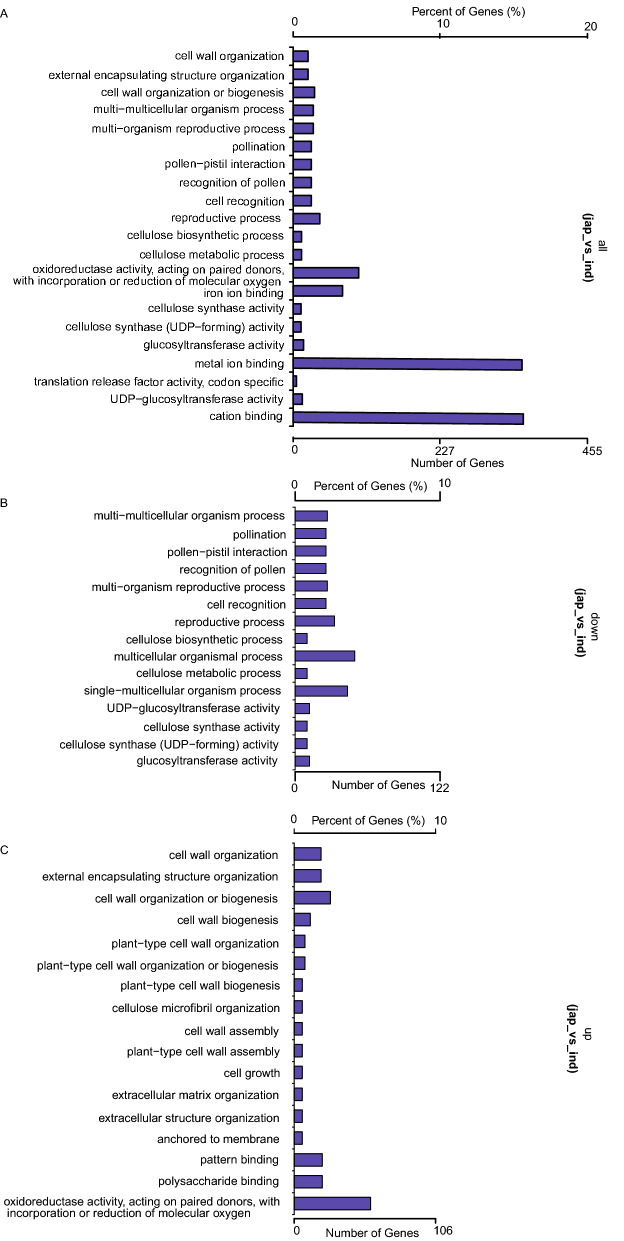
Histogram of Gene Ontology classifications. The letters associated with the x-axis indicate the GO categories, and the y-axis indicates the number of unigenes in each category. (**A**) GO analysis of all the DEGs. (**B**) GO analysis of the down-regulated genes. (C) GO analysis of the up-regulated genes. This figure was created using Goseq software.

We annotated the DEGs using enrichment analysis tool. According to the annotations, 21 Gene Ontology (GO) terms were significantly enriched (*P* < 0.05; Fig. 6). These terms could be classified into two groups. The first group consisted of reproduction-related terms, including “reproduction”, “recognition of pollen”, “multi-organism reproductive process” and “cell recognition”. The second group consisted of cell wall biosynthesis-related terms, including “cell wall organization”, “cellulose biosynthetic process”, “cellulose metabolic process”, “cellulose synthase activity” and “UDP-glucosyl transferase activity”. The GO analysis showed that the genes encoding the binding proteins were enriched in these co-expressed genes (Fig. 6). Interestingly, genes expressed at higher levels in *indica* rice than in *japonica* rice were annotated with reproduction-related terms, whereas those expressed at lower levels in *indica* rice than in *japonica* rice were annotated with cell wall biosynthesis-related terms. Consistent with these findings, among the ten most significant morphological differences between the two subspecies, six were reproduction-related traits.

To explore candidate pathways and genes that might be associated with the domestication of *indica* and *japonica* rice, we analyzed the enriched KEGG pathways (*P* < 0.05). Our results showed that the top four enriched pathways were regulation of autophagy (dosa04140), arginine and proline metabolism (dosa00330), pyrimidine metabolism (dosa00240), and starch and sucrose metabolism (dosa00500). It has been reported that the amylose content is usually higher in endosperm starch of *indica* rice than that of *japonica* rice^41^. In our results, there were 184 genes annotated being involved in the starch and sucrose metabolism pathway, with 15 DEGs (5 up- and 10 down-regulated genes in *japonica* rice) between the two subspecies. Overall, these results indicate that rice domestication has significant effects on starch and sucrose metabolism.

### Effect of selection pressure on the regulation of the DEGs

**Figure 7:**
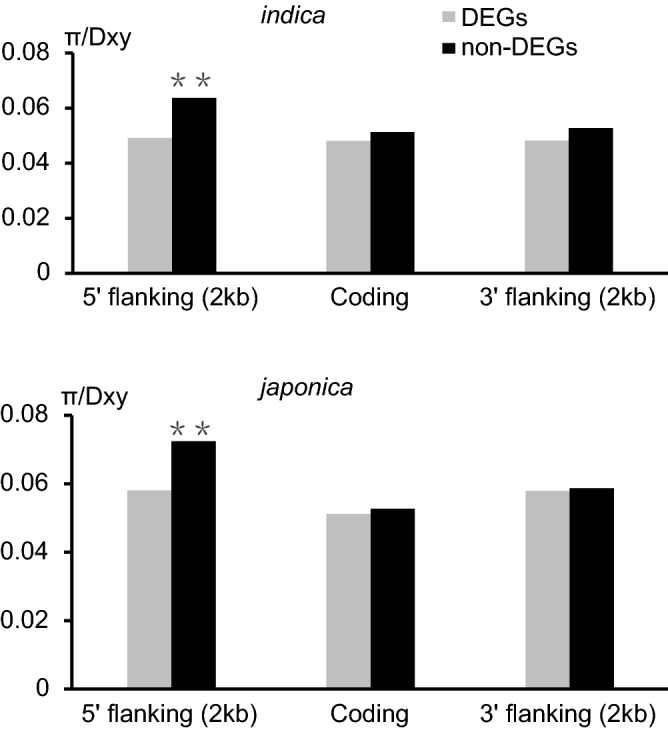
Polymorphism and divergence in gene-coding and regulatory regions of genes in *indica* and *japonica* rice. Y-axis indicates the ratio of polymorphism to divergence (π/Dxy), and bars in the x-axis represent the 5′ regulatory regions, gene-coding regions and 3′ regulatory regions of genes. The grey blocks indicate the DEGs, and the black blocks represent the non-DEGs. ** indicates P-value < 0.05. This figure was created using Microsoft Excel 2013 and Adobe Illustrator CS6.

To explore whether artificial selection may have affected the expression of the DEGs, we investigated the gene regulatory regions (i.e., 5′ and 3′ flanking regions) and gene-coding regions of the DEGs (Fig. 7, Table S6). Specifically, we searched for genomic regions with polymorphism to divergence (π/Dxy) ratios ranked in the lowest 5% (*P* < 0.001), because such regions may have a greater chance of experiencing positive selection. Compared with non-DEGs, DEGs from both subspecies had more 5′ flanking regions with low π/Dxy ratios (Fisher’s exact test, *P* < 0.05). These results were then confirmed by a resampling test (*P* < 0.05). Taken together, these results suggest that the 5′ regulatory regions of the DEGs experienced positive selection during rice domestication, and that gene regulation plays an important role in the evolution of the two subspecies.

## Discussion

In this study, the transcriptomes of two rice subspecies, *indica* and *japonica*, were characterized by the high-throughput RNA-Seq approach. The 31.58 billion RNA-Seq data provided us with valuable resources to discover novel transcriptionally active regions, identify DEGs and investigate the effect of selection pressure on the expression of the DEGs. According to the read mapping results, 25% of the clean reads were mapped to intergenic regions in the reference rice genome, suggesting the identification of 4,579 novel transcriptionally active regions. This speculation was then confirmed by RT-PCR, which validated the expression of some of these transcripts in all the samples tested. Therefore, in addition to characterizing the transcriptome of annotated rice genes, we also identified 4,579 novel transcriptionally active regions. More than 53% of these novel transcriptionally active regions may have been transcribed into ncRNAs because: (1) the transcribed fragments could be processed into mature transcripts with a mean length of 200 bp, which is the general length of long noncoding RNAs^42^; (2) 98.76% of the interval sequences between these transcribed regions were less than 5 kb in length, which is consistent with the distribution characteristics of intergenic pre-miRNAs^42^; and (3) the sequences of these transcribed regions shared high similarity with 37.2% of the ncRNAs deposited in the Ensembl ncRNA database. These results also highlight the significance of RNA-Seq in identifying the boundaries between sense-antisense gene pairs and quantitatively characterizing transcriptomes at a high resolution. Taken together, these results improve our understanding of the rice genome.

The expression patterns of different rice genes may vary during different developmental stages and in different plant individuals. Therefore, it is important to obtain RNA samples from a large number of plant individuals to reveal statistically significant differences in gene expression levels between rice subspecies. In this study, we obtained RNA samples from three young panicles of each of the 26 *indica* and 25 *japonica* accessions and searched for DEGs between the two rice subspecies based on the RNA-Seq data. In a previous study, the percentage of DEGs between closely related species in all detected genes varied greatly from 2 to 25%^43^. In extreme situations, this percentage can reach up to 78%^22^. The big differences in the percentage of DEGs among these studies may have been due to the fact that they use different gene expression quantification (e.g., qRT-PCR, microarray, and RNA-Seq) and data analysis methods^26^. Nonetheless, these studies have demonstrated that the percentages of DEGs between two species are generally higher than those between two subspecies. For example, a previous transcriptomic study found that 3.3% of the 18,242 genes detected were expressed at significantly different levels between maize and its progenitor teosinte^44^, whereas in another study, the percentage of DEGs between wild and weedy sunflowers (*Helianthus annuus*) was 5%^45^. In this study, we found that 3.3% of all the genes detected were expressed at significantly different levels between *indica* and *japonica* rice, suggesting that the strong artificial selection altered the expression of a small proportion of rice genes.

During artificial selection, cultivated rice has undergone significant changes in agronomically important traits, such as grain yield and traits that associated with abiotic stress resistance. We analyzed DEGs in depth and found that the expression levels of *GW2* (*LOC_Os02g14720*) and *OsBISERK1* (*LOC_Os08g07760*), which control grain width and length, respectively, were significantly different between the two rice subspecies. The expression of *GW2* was higher in *japonica* than in *indica*, whereas that of *OsBISERK1* was higher in *indica*. These results are in agreement with the grain shape of *indica* and *japonica* rice. Nitrogen is a crucial nutrient for plant growth and development. *Indica* has higher nitrate-absorption activity than *japonica*^46^. Here we show that the expression levels of *OsARG* (*LOC_Os04g01590*)^36^ and *OsNRT1* (*LOC_Os03g13274*)^37^ were both higher in *indica* than in *japonica*, which may contribute to this difference in nitrate use between these two subspecies. Due to the remarkable adaptability to different ecological and geographical environments, the two major rice subspecies, *indica* and *japonica* are distinct in the ability to tolerate cold stress. In general, *japonica* cultivars are more cold-tolerant than *indica*^47,48^. Proline-rich protein (PRP) genes, *OsPRP3* (*LOC_Os10g05750*), is positive regulators that control the cold tolerance^39^, Our RNA-seq data showed the expression levels of the two genes were much higher in *japonica* than in *indica* rice, which is consistent with the previous conclusion. Starch, which is composed of amylose and amylopectin, is the key determinant of rice quality. There are great differences in ECQs between *japonica* and *indica* during rice domestication^49,50^. In this study, three genes related to starch synthesis, *Wx* (*LOC_Os06g04200*), *OsSSI* (*LOC_Os06g06560*), and *OsAGPL1* (*LOC_Os03g52460*) had the same expression trend, with higher expression levels in *indica* rice, which potentially affect rice quality.

According to our GO analysis, genes that were expressed at lower levels in *indica* rice than in *japonica* rice were annotated with reproduction-related terms, whereas those that were expressed at higher levels in *indica* rice than in *japonica* rice were annotated with cell wall biosynthesis-related terms. Therefore, it is reasonable to speculate that differences in gene expression patterns are one of the reasons for the reproductive isolation between the two rice subspecies.

The contribution of genetic drift and selection pressures to the genetic and phenotypic differences between species has become a research hotspot in evolutionary biology in the past few decades. During rice domestication, artificial selection has been reported to have a major impact on rice gene expression^42^. According to previous studies on domesticated plants, the 5′ regulatory regions of many genes evolved under the pressure of artificial selection and that the 5′ regulatory regions of DEGs evolved faster those of non-DEGs^22^. Consistent with these results, multiple studies in model fish species found that nucleotide diversity in gene regulatory regions, rather than gene-coding regions, are important for speciation^22,51^. The relationships between nucleotide diversity and gene expression during species evolution have also been investigated by transcriptomic studies in Drosophila, fire ants, Arabidopsis and maize, and by comparative transcriptomic studies in humans and chimpanzees, and humans and mice. Taken together, these results support the hypothesis that gene regulatory regions and gene-coding regions evolve independently during speciation. Meanwhile, divergence in 5′ regulatory regions of DEGs is a strong indication for the involvement of positive selection during speciation. In this study we looked at the role of RNAs transcript in shaping the changes in cell wall biosynthesis and reproductive processes that occurred during rice domestication. We’ve found that they play an important role in this process. Although the research on crop domestication using genomics and genomic technology has been carried out for nearly 20 years, we still know very little about the genetic basis of most domestication traits in crop varieties. Early research tended to the simple traits controlled by only one or two genes and mutations could be easily identified. It is more difficult to identify more subtle developmental changes that are critical to many changes during the domestication. This research is a step by studying a regulatory mechanism that is essential for regulating changes related to rice domestication.

## Methods

### Sample collection and total RNA isolation

In a previous study, we collected seeds from 825 *Oryza sativa* ssp. *japonica* and *indica* accessions in a rice cultivation area and assessed the morphological and genetic differences between the two subspecies^5^. Based on this previous study, we selected 26 *indica* and 25 *japonica* accessions and cultivated them in an artificial climate chamber under conditions of 12 h light and 12 h dark (Table S1).

For total RNA isolation, three 40-mm-long young panicles were collected from each rice plant. These panicles were subjected to RNA isolation using TRIzol reagent (Invitrogen, USA), according to the manufacturer’s instructions. The purity and integrity of the RNA were determined by the NanoPhotometer® spectrophotometer (IMPLEN, USA) and the RNA Nano 6000 Assay Kit of the Bioanalyzer 2100 system (Agilent Technologies, USA), respectively. Thereafter, rRNA was removed from the total RNA samples and the rRNA-depleted total RNA was subjected to library construction using the NEBNext® Ultra™ Directional RNA Library Prep Kit for Illumina® (NEB, USA), according to the manufacturer’s instructions. First strand cDNA was synthesized using random oligonucleotides and SuperScript II. Second strand cDNA synthesis was subsequently performed using DNA Polymerase I and RNase H.

### RNA-Seq data filtering and assembly

Quality control of the raw data was performed with an in-house-developed PERL script. Low quality reads, reads with adaptor sequences and reads containing poly-N stretches were removed. The number of remaining clean reads for each sample is shown in Table S2. At the same time, Q20, Q30 and GC content of the clean reads were calculated (Table S2). To further evaluate the quality of the RNA-Seq data, we mapped reads from *japonica* and *indica* accessions to a reference *japonica* genome (RGAP 7.0) and a pseudo-transcriptome of *indica*^34^, respectively. The reference index was built using Bowtie v2.2.6^52^. In addition, paired-end clean reads were aligned to the reference genome with TopHat v2.1.0^53^. Duplicate reads were removed using SAMtools v1.2.5^54,55^ and Picard v1.39. All the results are summarized in Table S2.

The mapped reads of each sample were assembled using Scripture beta2^56^ and Cufflinks v2.1.1^57^. Cufflinks was also be used to estimate the expression of a transcript based on the mapping results^58^.

### Identification of DEGs

The FPKM of each gene was calculated using Cufflinks v2.1.1^57^. We performed principal variance component analysis to evaluate the differences in gene expression levels between the two subspecies, and the Pearson correlation coefficients between RNA-Seq data and RT-PCR results were also calculated. To identify a gene as differentially expressed, we used the Cuffdiff program within the Cufflinks. With the application of a model based on the negative binomial distribution^57^, Cuffdiff presents statistical routines for the determining of differential expression in the transcripts of gene expression data. To control the false discovery rate, we filtered out genes that were expressed at a level of less than 20 mapped reads in any of the 51 plants. We also calculated the Bayesian posterior P-value of each gene using a previously described method^59^.

### Determination of the effect of selection pressure based on resequencing data

To identify polymorphisms in all the DEGs and non-DEGs, we explored the resequencing data obtained from 12 *japonica*, 12 *indica* and 5 *O. rufipogon* plants grown in China^60^. The genomic DNA from the 29 individuals was sequenced at an 18–20 bp resolution and approximately 5.23 million single-nucleotide polymorphism (SNP) loci were obtained. We calculated the nucleotide diversity for *japonica* (πj), *indica* (πi) and *O. rufipogon* (πw) populations using Vcftools v0.1.13. A rank-based method was adopted to determine the nucleotide diversity in the 5′ flanking regions (2000 bp from start coden), gene-coding regions and 3′ flanking (2000 bp from stop coden) regions of the DEGs and non-DEGs between *O. rufipogon* and *japonica* or between *O. rufipogon* and *indica*. The results were confirmed by a resampling test, which was repeated 1000 times.

### GO and KEGG analysis

To annotate the functions of the DEGs, we performed Gene Ontology (GO) analysis using a GO Seq R package, in which the gene length bias was corrected. We also calculated the FDR-corrected P-values for the GO terms using hypergeometrical distribution. GO terms with corrected P-values less than 0.05 were considered to be significantly enriched. KEGG is a database resource for understanding genome sequencing and other high-throughput experimental technologies (http://www.genome.jp/kegg/)^61,62^. We used KOBAS software to test the statistical enrichment of DEGs in KEGG pathways.

### Quantitative RT-PCR analysis

Quantitative RT-PCR was performed using the SYBR® Premix Ex TaqTM Kit (TaKaRa, Japan) on an ABI PRISM 7900HT platform (Applied Biosystems, USA), according to the manufacturer’s instructions. For each sample, two biological replicates were analyzed by three technical repeats. The rice *ubiquitin-1* gene (*LOC_Os03g13170*) was used as the internal reference gene. The primers for qRT-PCR analysis are listed in Table S5.

## Supplementary Information

Supplementary Information 1.

Supplementary Information 2.

Supplementary Information 3.

Supplementary Information 4.

Supplementary Information 5.

Supplementary Information 6.

## Data Availability

All the data generated or analyzed during this study are included in the published version of this paper and its supplementary information files. The sequences have been deposited into the NCBI Sequence Read Archive database (PRJNA 610458 and PRJNA 628160).
